# The Effect of Guidance regarding Home Exercise and ADL on Adolescent Females Suffering from Adverse Effects after HPV Vaccination in Japanese Multidisciplinary Pain Centers

**DOI:** 10.1155/2016/3689352

**Published:** 2016-04-18

**Authors:** Takahiro Ushida, Masahiko Shibata, Masaki Kitahara, Shoji Yabuki, Masahiko Sumitani, Takanori Murakami, Masako Iseki, Masako Hosoi, Hiroaki Shiokawa, Tomoko Tetsunaga, Hiroyuki Nishie, Sei Fukui, Motohiro Kawasaki, Sinsuke Inoue, Makoto Nishihara, Shuichi Aono, Tatunori Ikemoto, Takashi Kawai, Young-Chang Arai

**Affiliations:** ^1^Multidisciplinary Pain Centre, School of Medicine, Aichi Medical University, Aichi 480-1195, Japan; ^2^Department of Pain Medicine, Osaka University, Graduate School of Medicine, Japan; ^3^Pain Clinic, The Jikei University Hospital, Japan; ^4^Department of Orthopedic Surgery, Fukushima Medical University School of Medicine, Japan; ^5^Department of Pain and Palliative Medicine, The University of Tokyo Hospital, Japan; ^6^Department of Rehabilitation Medicine, Sapporo Medical University, Japan; ^7^Department of Anesthesiology and Pain Management, Juntendo University School of Medicine, Japan; ^8^Department of Psychosomatic Medicine, Kyushu University Hospital, Japan; ^9^Department of Anesthesiology & Critical Care Medicine, Kyushu University Hospital, Japan; ^10^Department of Orthopedic Surgery, Okayama University Hospital, Japan; ^11^Department of Anesthesiology and Resuscitology, Okayama University Hospital, Japan; ^12^Interdisciplinary Pain Management Center, Shiga University of Medical Science Hospital, Japan; ^13^Department of Orthopedic Surgery, Kochi Medical School, Japan

## Abstract

*Background*. Two prophylactic papillomavirus (HPV) vaccines have been available for primary prevention of cervical cancer. Although serious adverse effects (AE) were rare, more than 230 women have been suffering from severe AEs such as persistent pain and headache in Japan. Our research group started to treat adolescent females suffering from the AEs.* Objective*. To survey the characteristics of and the effects of cognitive behavioral therapy on adolescent female suffering from the AEs in Japanese multidisciplinary pain centers.* Methods*. One hundred and forty-five patients suffering from the AEs were reviewed retrospectively and 105 patients of them were provided guidance on home exercise and activities of daily living based partially on a cognitive-behavioral approach. The intensity of pain was rated by the patients using a numerical rating scale (NRS). Furthermore, the Hospital Anxiety and Depression Scale (HADS) and the Pain Catastrophizing Scale (PCS) were used.* Results*. Eighty out of the 105 patients who received the guidance were followed up, 10 displayed a marked improvement, and 43 showed some improvement.* Conclusions*. Guidance on home exercise and activities of daily living based on a cognitive-behavioral approach alleviated the AEs that women suffered from after HPV vaccination in Japan.

## 1. Introduction

Cervical cancer is the fourth most common cancer in women in the world [1]. Genital infection with human papillomavirus (HPV) types 16 and 18 can cause cervical cancer [2]. Two prophylactic HPV vaccines (quadrivalent and bivalent) have been available for primary prevention of cervical cancer in over 100 countries. Safety of both vaccines has been evaluated [2, 3]. Local and systemic injection-related symptoms were generally mild. Serious adverse effects (AEs) were rare. The most common local AEs are injection site pain, injection site swelling, and injection site erythema. The most systemic AEs are vasovagal syncope, dizziness, nausea, and headache. Based on these reports, the event rate was less than 0.1%.

Both vaccines were introduced in Japan in 2010 and Japanese women were required to receive one of these vaccines from April 2013. The introduction of the vaccines has generated great controversy over its safety in Japan [4]. In Japan, 8.75 million doses were used. Mass media have reported that more than 230 women have been suffering from severe AEs such as persistent pain and headache after vaccination. Vaccine Adverse Event Reporting System in the United States received 25,176 adverse events following HPV vaccination: a rate of serious adverse events was 2-3 reports per 100,000 doses [2, 5]. Although the rate of serious adverse events in Japan was the same as that of the United States, several doctors, the social community of these girls and family members, and mass media have challenged the recommendation, so Japan could not overlook the status and has stopped recommending the HPV vaccines. In Japan, some researchers regard the symptoms as a consequence of psychosomatic reactions, while others propose alternative plausible mechanisms. The Ministry of Health, Labor and Welfare asked us to organize a research group in order to treat women suffering from AEs after HPV vaccination. Our research group started to treat women suffering from AEs since July 2013. In almost all cases, there was no clinical or laboratory evidence of relevant pathology in their symptoms despite insistent complaints including headache and musculoskeletal pain.

Chronic pain of adolescents interferes with their daily lives and is associated with functional disability and distress [6, 7]. In these cases, satisfactory treatment outcomes may be unachievable with a uniform therapeutic approach seeking only to eliminate pain. Often a multifaceted, comprehensive approach is needed. In particular, therapeutic approaches based on cognitive-behavioral therapy (CBT) under multiple academic disciplines (multidisciplinary) are recommended [6–9]. Pain and function improve in youth receiving CBT for chronic pain [6, 7]. CBT consists of behavioral strategies for engagement with normal daily activities, a focus on the self-regulation of emotion and the use of technique for reducing aversive arousal. Thus, we decided to consider these women as common patients suffering from chronic pain and treated them by providing guidance on home exercise and activities of daily living (ADL) based partially on a cognitive-behavioral approach under multiple academic disciplines. We report here the characteristics of and the effects of cognitive-behavioral therapy on women suffering from AEs after HPV vaccination in Japanese multidisciplinary pain centers.

## 2. Methods

### 2.1. Samples and Informed Consent

Retrospective analysis from July 2013 to July 2014 was performed on women who visited multidisciplinary pain centers (Pain Centers) in Japan. All patients were referred from other hospitals to the Pain Centers because of AEs after HPV vaccination. Treatment protocols used in the present report were based on institutional policy and clinical guidelines approved by the IRB of each institution. After obtaining approval from the IRB (reference number of Aichi Medical University: 12-067), we routinely explain to all patients that we record and store demographics, symptoms, course of pain, and medical records of all patients for future possible use in our research. After we explained these details, we obtained written informed consent upon their initial visit to the Pain Centers.

## 3. Procedures

### 3.1. Interview

We saw the patients and checked their clinical and laboratory data which they brought to the center. We sometimes performed additional medical examinations if the data they provided were insufficient. Based on their symptoms, signs, physical condition, and medical data, we confirmed and explained to these women that in almost all cases there was no clinical or laboratory evidence of relevant pathology in their pain and its associated symptoms. Some patients and family members declined to accept this (*n* = 40). Three-quarters of the women who presented (*n* = 105) accepted this, to whom we proposed the provision of guidance on home exercise and ADL based partially on a cognitive-behavioral approach.

### 3.2. Measures

An assessment battery of standardized self-report measures, demographics, symptoms, history, and duration of pain was administered to all patients at the initial visit. The intensity of pain was rated by the patients using a numerical rating scale (NRS) where 0 indicated no pain and 10 the greatest pain possible. Furthermore, we used the Hospital Anxiety and Depression Scale (HADS) to determine the levels of anxiety and depression and the Pain Catastrophizing Scale (PCS) to determine the levels of physical and emotional distress associated with their current pain problems that they and family members associated with their HPV vaccination. The HADS consists of 14 items; the anxiety (HADS-A) and depression (HADS-D) subscales each include 7 items [9–11]. A 4-point response scale (from 0 representing absence of symptoms, to 3 representing maximum symptoms) is used, with possible scores for each subscale ranging from 0 to 21. Moreover, the PCS is a 13-item scale that assesses three types of negative thinking styles related to pain [11–13]. Subjects are asked to reflect on past painful experiences and indicate on a 5-point scale ranging from 0 (“not at all”) to 4 (“always”) the degree to which they experienced each of the 13 thoughts or feelings when in pain.

### 3.3. Treatments

We gave them and family members some explanations as follows: prolonged pain can result in sleep disorders, decreased desire, anxiety, depression, and decreased routine activity, occasionally causes withdrawal from school or society, and otherwise disrupts daily activities [6, 7, 9, 14]. As a result, patients with chronic pain fall into a vicious circle in which these psychological and social factors complicate their condition [6–9]. In such cases, therapeutic approaches based on CBT under multiple academic disciplines (multidisciplinary) are recommended. CBT makes patients deepen their understanding of their pain and teaches self-control and coping strategies in order to encourage behavioral modifications that allow them to better confront their pain and maintain and improve ADL as well [6–9, 14, 15].

Furthermore, the guidance was designed to inform the patients and family members about how to correct or eliminate excessive fear of pain, improper thinking for treating pain, and anxiety caused by distorted cognition as well as how to control activity levels by appropriate pacing by a doctor (anesthesiologist or orthopedic surgeon) for engagement with normal daily activities and the patients and family members were asked to do these matters at home. The guidance was arranged according to their daily activities at each visit. Frequency of visits ranged from once a month to once every two months. For stretching, a doctor (orthopedic surgeon) or physiotherapist instructed the patients to stretch the muscle groups in their shoulder girdles, lumbar area, hips, thighs, and lower legs tailored to each female. Stretching consisted of self-performed static stretching with the muscle groups extended for 20 seconds each twice a day. Also, they were instructed in muscular strengthening exercises to strengthen the trunk and leg muscle groups in the supine, sitting, and standing positions, performing 10 repetitions under their own body weight tailored to each female twice a day. In 9 cases, psychotherapists provided the females and their family members with counseling as needed.

### 3.4. Data Analysis

Patients were categorized as showing marked signs of improvement (≥60% improvement in numeric rating scale (NRS) for pain compared to initial visit), some improvement (>20%–60% improvement in NRS compared to initial visit), no improvement (0%–<20% improvement in NRS compared to initial visit), and deterioration (<0% improvement in NRS compared to initial visit) depending on the state of pain 6 months after the initial visit. Moreover, patients who did not visit the hospital again even with an appointment for a follow-up visit and those who visited the Pain Centers to receive a second opinion were categorized separately.

## 4. Results

The characteristics of the patients are described in Table 1. There was no significant difference between the accept and decline groups in the type of vaccine (Chi-squared test, *p* = 0.1443). There was no significant difference between the two groups in the vaccine doses (Chi-squared test, *p* = 0.9366). There was no statistically significant difference between the two groups in the median (range) duration of symptoms (Mann-Whitney test, *p* = 0.2839). The median (range) of anxiety subscale of pretreatment HADS was 5 (0–17) in the accept group and 6 (0–15) in the decline group (Mann-Whitney test, *p* = 0.2140). The median (range) of depression subscale of pretreatment HADS was 4 (0–16) in the accept group and 5 (0–16) in the decline group (Mann-Whitney test, *p* = 0.2409). The median (range) of pretreatment PCS was 27.5 (0–51) in the accept group and 30 (0–52) in the decline group (Mann-Whitney test, *p* = 0.8238). NRS showed a statistically significant difference but the difference was not clinically significant.

Table 2 shows the distribution of symptoms in the accept group for each age. There were no significant differences of the distribution of symptoms in line with the age of the subjects (Chi-squared test, *p* = 0.1807). Pain improvement in the accept group was described for each age in Table 3. There were no significant differences of the distribution of the improvement in line with the age of the subjects (Chi-squared test, *p* = 0.5521). There were no significant differences in treatment outcomes for each different type of symptom (Chi-squared test, *p* = 0.6826) (Table 4). There were no significant differences of duration of symptom and pretreatment HADS and PCS scores among the treatment outcomes (Table 5).

## 5. Discussion

Two prophylactic HPV vaccines (quadrivalent and bivalent) were introduced in Japan. The cause of serious symptoms among teenage girls vaccinated with the HPV vaccines and their safety have yet to be verified. In the present survey, we confirmed and explained to these women that in almost all cases there was no clinical or laboratory evidence of relevant pathology in their pain and its associated symptoms, based on their symptoms, signs, physical condition, and medical data. While some patients and family members declined to accept this (*n* = 40), three-quarters of the women who presented (*n* = 105) accepted this. In Japan, some researchers regard the symptoms as a consequence of psychosomatic reactions, so we had expected that psychological issues of the decline group might have been worse than those of the accept group. Although we compared the background between these two groups, we did not identify any significant differences in the type and dose of vaccine, the duration of symptoms, and psychological issues of HADS and PCS. However, we think that we need further studies in these points. NRS showed a statistically significant difference but we considered that the difference was not clinically significant.

Given that in almost all cases there was no clinical or laboratory evidence of relevant pathology in their pain, we handled these women as common patients suffering from chronic pain and treated them with the provision of guidance on home exercise and activities of daily living based on a cognitive-behavioral approach under multiple academic disciplines [6–9]. As shown in this case series, this kind of approach improved the symptoms in 66.7% of the 80 patients who were followed up. Although we thought that some of the symptoms experienced by participants might have been influenced by stage of mental and physical development and endocrine factors that change while women develop into reproductive phases, the present results did not show that kind of influence. And we found most treatment responders showed moderate rather than marked improvement. We also analysed types of symptoms associated with the treatment outcomes and we did not find out the significant differences, but myalgia sufferers were less likely to experience pain reduction. We thus postulate that these sufferers easily experience somatization and it might be hard for them to realise the improvement of their symptoms. Moreover, we analysed psychological issues associated with the treatment outcomes and we did not find out the significant differences in this survey. However, we need further studies in these areas.

There are several limitations to this study. First, the present report is a retrospective case series and not a randomized control analysis, but AEs after HPV vaccination have been an object of public concern. Thus, we thought that we should not have treated women suffering from AEs based on randomized control research in the Pain Centers. We did not focus on the cause of serious symptoms among teenage girls vaccinated with the HPV vaccines and their safety in this case series. We thus need further research by comparing women who suffer from AEs with women who do not, in order to ascertain the cause of serious symptoms after HPV vaccination and its safety. Also, we need to clarify what components of treatment are associated with symptom improvement and whether improvement associated with different symptoms differs with treatment component.

In conclusion, guidance on home exercise and activities of daily living based on a cognitive-behavioral approach alleviated, to some extent, the AEs that women suffered from after HPV vaccination in Japan.

## Additional Points

Two prophylactic papillomavirus (HPV) vaccines (quadrivalent and bivalent) have been available for primary prevention of cervical cancer. More than 230 women have been suffering from severe adverse effects (AEs) such as persistent pain and headache after vaccination in Japan, so Japan has stopped recommending the HPV vaccines. Our research group started to treat women suffering from AEs after HPV vaccination. Guidance on home exercise and activities of daily living based on a cognitive-behavioral approach alleviated, to some extent, the AEs that adolescent females suffered from after HPV vaccination in Japan.

## Figures and Tables

**Table 1:** Patients' characteristics.

	Accept	Decline	*p*
Age (years), median (range)	15 (12–19)	16 (12–19)	0.0766
Vaccine, *n* (%)			0.1443
Quadrivalent	33 (31.4)	9 (25.0)	
Bivalent	69 (65.7)	27 (65.0)	
Unknown	3 (2.8)	4 (10.0)	
Vaccine doses, *n* (%)			0.9366
3 doses	77 (73.3)	31 (77.5)	
2 doses	17 (16.2)	5 (12.5)	
1 dose	9 (8.6)	3 (7.5)	
Unknown	2 (1.9)	1 (2.5)	
Onset			0.5483
After first vaccination, *n* (%)	9 (8.6)	2 (5.0)	
Onset interval (days), median (range)	0 (0–21)	10 (9–11)	
After second vaccination, *n* (%)	13 (12.4)	7 (17.5)	
Onset interval (days), median (range)	1 (0–90)	9 (0–90)	
After third vaccination, *n* (%)	65 (61.9)	22 (55.0)	
Onset interval (days), median (range)	30 (0–1230)	105 (1–690)	
Duration of symptom (months), median (range)	12 (1–48)	18 (2–37)	0.2839
Pretreatment HADS			
Anxiety, median (range)	5 (0–17)	6 (0–15)	0.2140
Depression, median (range)	4 (0–16)	5 (0–16)	0.2409
Pretreatment PCS, median (range)	27.5 (0–51)	30 (0–52)	0.8238
Pretreatment NRS of pain, median (range)	5 (0–10)	4 (0–7)	0.0207^*∗*^

HADS, Hospital Anxiety and Depression Scale. PCS, Pain Catastrophizing Scale. NRS, numerical rating scale. ^*∗*^Significant difference.

**Table 2:** Symptoms of 105 patients in the accept group.

	*n*, *p* = 0.1807							
Age	12	13	14	15	16	17	18	19
Headache	2	0	3	7	1	2	2	0
Myalgia	0	5	7	3	3	2	3	1
Arthralgia	0	4	7	2	2	2	0	2
Dizziness	0	0	1	0	0	1	1	0
More than one symptom	1	6	14	8	7	5	0	1

**Table 3:** Treatment outcomes in the accept group.

	*n*, *p* = 0.5521							
Age	12	13	14	15	16	17	18	19
Marked improvement	1	2	4	1	1	0	1	0
Some improvement	0	8	12	7	6	5	3	2
No improvement	1	3	7	6	4	3	1	0
Deterioration	0	0	0	0	2	0	0	0

**Table 4:** Treatment outcomes for each different type of symptom in the accept group.

	*n*, *p* = 0.6826				
Symptom	Headache	Myalgia	Arthralgia	Dizziness	More than one symptom
Marked improvement	4	1	1	0	4
Some improvement	7	7	11	2	16
No improvement	3	7	3	1	9
Deterioration	1	0	0	0	1

**Table 5:** Treatment outcomes and duration of symptom, HADS, and PCS in the accept group.

	Median (range)			
	Duration of symptom	HADS		PCS
	Months	Anxiety	Depression	
Marked improvement	11 (1–31)	5 (1–9)	5 (0–13)	27 (18–36)
Some improvement	9.5 (1–40)	4 (0–11)	3 (0–14)	30 (6–42)
No improvement	9 (1–49)	5 (0–15)	5 (0–15)	27 (0–45)
Deterioration	8.5 (5–12)	3.5 (1–6)	5.5 (4–7)	24 (12–36)
*p* (Kruskal-Wallis test)	0.1139	0.4978	0.4098	0.9650

HADS, Hospital Anxiety and Depression Scale. PCS, Pain Catastrophizing Scale.
